# Bleeding Complications Associated with Pregnancy with Primary Immune Thrombocytopenia: A Meta-Analysis

**DOI:** 10.1055/a-1837-7581

**Published:** 2022-08-29

**Authors:** Jose Ramon Gonzalez-Porras, Danylo Palomino, Luis Mario Vaquero-Roncero, Jose María Bastida

**Affiliations:** 1 Department of Hematology, Instituto de Investigación Biomédica de Salamanca (IBSAL), Complejo Asistencial Universitario de Salamanca (CAUSA), Universidad de Salamanca (USAL), Salamanca, Spain; 2 Department of Anesthesiology, Reanimation and Pain Medicine, Instituto de Investigación Biomédica de Salamanca (IBSAL), Complejo Asistencial Universitario de Salamanca (CAUSA), Universidad de Salamanca (USAL), Salamanca, Spain

**Keywords:** primary immune thrombocytopenia, pregnancy, platelet count, maternal thrombocytopenia, neonatal thrombocytopenia, maternal bleeding, postpartum hemorrhage, intracranial hemorrhage

## Abstract

**Introduction** Immune thrombocytopenia (ITP) during pregnancy has received little attention from researchers. Reliable information about the outcome of mothers and newborns is required to properly counsel women who are pregnant or planning to become pregnant. Our primary outcomes were the frequency and severity of maternal and neonatal bleeding events in the setting of ITP in pregnancy. Mode of delivery, neonatal thrombocytopenia, and maternal/infant mortality were secondary outcomes.

**Material and Methods** We comprehensively reviewed the prospective studies that enrolled ≥20 pregnant women with primary ITP. Two reviewers, blinded to each other, searched Medline and Embase up to February 2021. Meta-analyses of the maternal and newborn outcomes were performed. Weighted proportions were estimated by a random-effects model.

**Results** From an initial screening of 163 articles, 15 were included, encompassing 1,043 pregnancies. The weighted event rate for bleeding during pregnancy was 0.181 (95% confidence interval [CI], 0.048–0.494). Most of these were nonsevere cases. The weighted event rates were 0.053 (95% CI, 0.020–0.134) for severe postpartum hemorrhage, 0.014 (95% CI, 0.008–0.025) for intracerebral hemorrhage, and 0.122 (0.095–0.157) for severe thrombocytopenia events in neonates (platelet count <50,000/μL). There were no reliable predictors of severe neonatal thrombocytopenia. The incidence of neonatal mortality was 1.06%. There were no maternal deaths.

**Conclusion** Primary ITP in pregnant women is rarely associated with poor outcomes.

## Introduction

Immune thrombocytopenia (ITP) is an immune-mediated bleeding disorder characterized by a reduced number of circulating platelets and an increased risk of bleeding. 1 It is caused by humoral and cell-mediated attacks on circulating platelets and bone marrow-resident megakaryocytes. 2 The disease accounts for 1 to 4% of the cases of pregnancy-associated thrombocytopenia, 3 4 which occurs in around 7 to 11% of pregnant women. 5 6 The usual bleeding phenotype of mothers is supposed to be mild. Indeed, not all mothers have to be administered immunosuppressant therapy. 7 8 Maternal ITP can result in neonatal thrombocytopenia, which, in turn, may be associated with bleeding complications. The most concerning of these is intracranial hemorrhage (ICH). 9 10

The management of ITP in pregnancy remains a challenge. 11 12 The relative rarity of the condition has precluded the design of large studies to find out the most suitable therapeutic decision in each particular situation. This scarcity of reliable analyses also makes it difficult to form an accurate perspective on how ITP influences maternal and neonatal outcomes. For this reason, we performed a systematic search to identify the studies on primary ITP, that is, ITP that is not associated with underlying infection/diseases. We performed a meta-analysis of the selected studies to shed light on the extent to which the following objectives had been realized. The primary objective was to describe the frequency and severity of maternal and neonatal bleeding events in the setting of ITP in pregnancy. The secondary objective was to describe the frequency of cesarean births, severe thrombocytopenia in neonates, and maternal/infant mortality.

## Materials and Methods

### Article Search

To select the eligible studies whose results would be included in the meta-analysis, two reviewers (D.M.P. and J.R.G.P.) independently searched the PubMed and Embase databases. The chosen keyword combinations were thrombocytopenic purpura OR immune thrombocytopenia OR ITP OR werlhof* disease AND pregnancy OR pregnant women. The most recent date included was February 24, 2021. Papers published before 1990 were excluded to ensure as much consistency as possible among the diagnostic procedures, and the platelet count and bleeding assessment methods. The reviewers both followed the same steps to obtain the definitive selection: examination of all references obtained by applying the search algorithms, exclusion of studies not relevant to the objectives of the review, and exclusion of relevant works that did not meet the inclusion and exclusion criteria set out below. Once the reviewers had finished their separate selections, they exchanged their set of references with the other reviewer, who had been blinded to the other's work until that moment. In the event that an article was selected by one reviewer only, both of them sought to reach a consensus. Only studies whose inclusion was agreed upon by both reviewers were included in the final selection.

### Inclusion and Exclusion Criteria

Selected studies had to follow a prospective design and assess at least two of the following: maternal thrombocytopenia, maternal bleeding during the antenatal period, type of labor; maternal bleeding at delivery/postpartum, neonatal ICH and other neonatal bleeding complications, neonatal thrombocytopenia, and neonatal and maternal mortality. The following were considered to be reasons for exclusion: non-English language, publication date earlier than 1990, patient cohort size smaller than 20 subjects, nonprospective design, and secondary ITP cases included in the cohort.

### Information Collected and Severity Criteria

The following information was looked for and extracted from the selected articles: aims of the study, number of pregnant women and newborns included, bleeding assessment tool (BAT) used, if any, maternal bleeding in pregnancy or at delivery/postpartum, mode of delivery, neonatal ICH and other neonatal bleedings, nadir of neonatal thrombocytopenia and predictor factors of this complication, and neonatal and maternal death. Severe neonatal thrombocytopenia was defined as a platelet count less than 50,000/μL. Severe postpartum hemorrhage (PPH) was defined as blood losses of ≥1,000 mL from the genital tract in the first 24 hours after delivery. 13

### Statistical Analysis

For each of the variables analyzed, weighted proportions were estimated by meta-analysis in a random-effects model. Results were reported as weighted event rates and their 95% confidence intervals (CIs). The Comprehensive Meta-Analysis Version 3 (Biostat Inc., Englewood, NJ, United States) program was used.

## Results

**Fig. 1:**
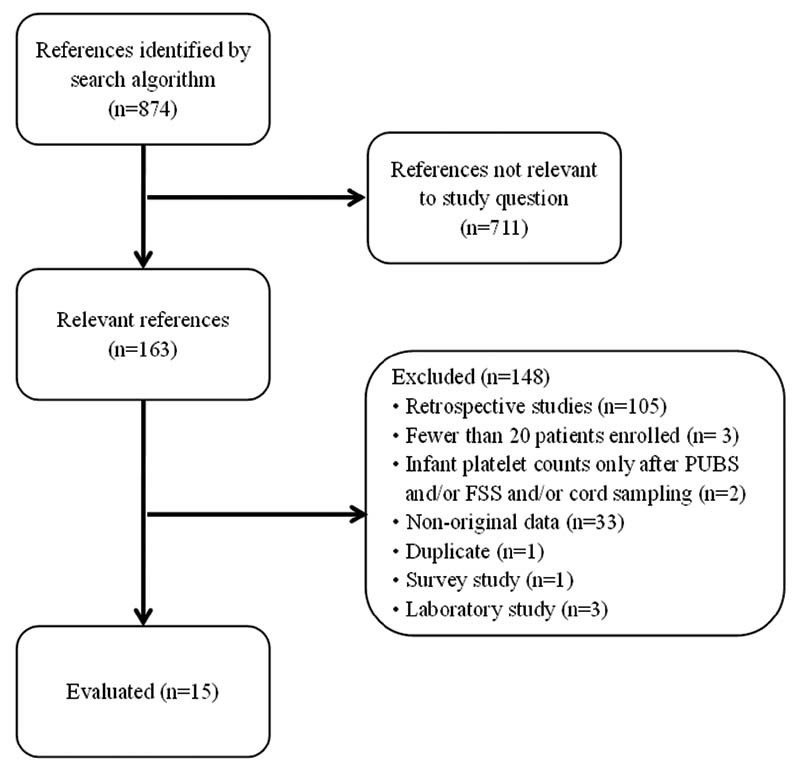
Flow diagram for selection of studies.

The search performed by following the criteria explained above allowed us to identify 874 articles, of which 711 were discarded because they did not address topics relevant to our study. The remaining 163 papers were carefully examined, leading us to exclude 148 of them for a variety of reasons, of which following a retrospective design was by far the most common ( *n* = 105). The final selection comprised 15 articles ( *n* = 1,043 pregnancies) ( Fig. 1 ). 14 15 16 17 18 19 20 21 22 23 24 25 26 27 28 Table S1 (available in the online version) depicts the chosen studies and includes information concerning their aims and the topics addressed. Among the variables addressed by this review, only the maternal and neonatal mortality were addressed in all the 15 selected articles. The number of studies included in the meta-analysis of the other items ranged between 6 and 14.

### Maternal Bleeding during Pregnancy

Six studies addressed maternal bleeding during pregnancy, 14 16 17 18 20 25 in which bleeds, irrespective of severity, were reported in 8 to 54% of cases ( Table 1 ). Most bleeding episodes were minor. The only study that used a BAT to evaluate severity ( Table S1 ) found that bleeding was mild/moderate in 93% of 31 patients, with ecchymoses, dripping with moderate loss of blood and, especially, petechiae. 18 Two studies that aimed to report only severe episodes did not document any cases from a total of 109 pregnancies. 20 25 Taking all six studies together, 113 bleeding events were documented from a total of 482 pregnancies. The weighted event rate for bleeding was 0.181 (95% CI, 0.048–0.494).

**Table 1:** Bleeding during pregnancy in mothers with ITP

*N*	Author	Year	Ref.	Pgn/Bb ( *n* / *n* )	Bleeding during pregnancy, *n*	Event rate (95% CI)
1	Wegnelius et al	2018	14	75/76	18 a	0.240 (0.157–0.349)
2	Care et al	2018	16	107/108	9 b	0.037 (0.014–0.095)
3	Rezk et al	2018	17	160/155 c	86	0.538 (0.460–0.613)
4	Kong et al	2017	18	31/31	31 d	0.984 (0.794–0.999)
5	Gandemer et al	1999	20	46/46	0 e	0.011 (0.001–0.149)
6	Mazzucconi et al	1993	25	63/63	0 e	0.008 (0.000–0.113)
Total	–	–		482/469	113 f	0.181 (0.048–0.494)

Abbreviations: Bb, babies; CI, confidence interval; ITP, immune thrombocytopenia; N, number of study, from most recent to oldest; Pgn, pregnancies; Ref., reference number.

a Eight mucosal bleeds, five vaginal bleeds, two petechiae, two nose bleeds, and one bruise.

b Four bruising, three gingival bleeds, and two purpura.

c There were five intrauterine fetal demises.

d Bleeding in pregnancy was one of the inclusion criteria and was graded according to the bleeding assessment tool reported by the Gruppo Italiano Malattie EMatologiche dell'Adulto ITP Working Party [28], with the following results: 0, *n* = 0 (0%); 1, *n* = 19 (61.3%); 2, *n* = 10 (32.3%); 3, *n* = 2 (6.4%); 4, *n* = 0 (0%).

e Data correspond to severe bleeding only (severity criteria were not specified).

f The studies of Kong, Gandemer, and Mazzucconi were not included in this calculation, the first because maternal bleeding was one of the inclusion criteria and the other two because only severe bleeding episodes were documented.

### Postpartum Hemorrhage

Four of seven studies that provided information about severe PPH did not document episodes among a total of 179 pregnant women with ITP 18 20 23 25 ( Table 2 ). In the other three cohorts, the incidence ranged between 5 and 20%. 14 16 19 The weighted event rate for severe postpartum hemorrhage was 0.053 (95% CI, 0.020–0.134).

**Table 2:** Severe postpartum hemorrhage in mothers with ITP

*N*	Author	Year	Ref.	Pgn/Bb ( *n* / *n* )	Severe PPH, *n*	Event rate (95% CI)
1	Wegnelius et al	2018	14	75/76	8	0.107 (0.054–0.199)
2	Care et al	2018	16	107/108	22	0.206 (0.139–0.293)
3	Kong et al	2017	18	31/31	0	0.016 (0.001–0.206)
4	Yassae et al	2012	19	21/20	1	0.048 (0.007–0.271)
5	Gandemer et al	1999	20	46/46	0	0.011 (0.001–0.149)
6	Yamada and Fujimoto	1994	23	39/41	0	0.013 (0.001–0.171)
7	Mazzucconi et al	1993	25	63/63	0	0.008 (0.000–0.113)
Total	–	–		382/385	31	0.053 (0.020–0.134)

Abbreviations: Bb, babies; CI, confidence interval; ITP, immune thrombocytopenia; *N* , number of study, from most recent to oldest; Pgn, pregnancies; PPH, postpartum hemorrhage; Ref., reference number.

### Intracranial Hemorrhage

Neonatal ICH was determined in 14 articles ( Table 3 ). Diagnoses were made based on ultrasonography. In two of the studies, diagnoses were performed serially in all infants born to ITP mothers, 23 24 while in the other twelve the procedure was undertaken only when there was clinical suspicion. Only three of the studies documented cases of ICH. In the first study, both infants presented with a platelet count of 5,000/μL, and the event occurred after vaginal delivery in one case and after cesarean section in the other. 22 In the second study, one infant, delivered per vaginum, presented with an intracranial hematoma with a platelet count of 6,000/μL. 26 In the third study, two cases of ICH, with platelet counts of 7,000 and 78,000 platelets/μL, were documented in two vaginally delivered infants, the former of the two having a fatal outcome. 28 Overall, there were five cases of ICH among 887 infants. The weighted event rate for IHC was 0.014 (95% CI, 0.008–0.025).

**Table 3:** Bleeding events in neonates of mothers with ITP

*N*	Author	Year	Ref.	Pgn/Bb ( *n* / *n* )	ICH, *n*	Event rate (95% CI)	Non-ICH, *n*	Event rate (95% CI)
1	Wegnelius et al	2018	14	75/76	0	0.006 (0.000–0.095)	1 a	0.013 (0.002–0.088)
2	Xu et al	2018	15	87/86	0	0.006 (0.000–0.085)	N.R.	–
3	Care et al	2018	16	107/108	0	0.005 (0.000–0.070)	N.R.	–
4	Kong et al	2017	18	31/31	0	0.016 (0.001–0.206)	0	0.016 (0.001–0.206)
5	Yassaee et al	2012	19	21/20	0	0.024 (0.001–0.287)	0	0.024 (0.001–0.287)
6	Gandemer et al	1999	20	46/46	0	0.011 (0.001–0.149)	5 b	0.109 (0.046–0.236)
7	Valat et al	1998	21	64/64	0	0.008 (0.000–0.111)	12 c	0.188 (0.110–0.302)
8	Christiaens et al	1997	22	68/68	2	0.029 (0.007–0.110)	N.R.	–
9	Yamada and Fujimoto	1994	23	39/41	0	0.012 (0.001–0.164)	N.R.	–
10	Burrows and Kelton	1993	24	46/46	0	0.011 (0.001–0.149)	N.R.	–
11	Mazzucconi et al	1993	25	63/63	0	0.008 (0.000–0.113)	4 d	0.063 (0.024–0.157)
12	Moutet et al	1990	26	32/32	1	0.031 (0.004–0.191)	0	0.015 (0.001–0.201)
13	Christiaens et al	1990	27	28/28	0	0.017 (0.001–0.223)	5 e	0.179 (0.076–0.364)
14	Samuels et al	1990	28	176/178	2	0.011 (0.003–0.044)	8 f	0.045(0.030–0.101)
Total	–	–		883/887	5	0.014 (0.008–0.025)	35	0.075 (0.041–0.133)

Abbreviations: Bb, babies; CI, confidence interval; ICH, intracranial hemorrhage; ITP, immune thrombocytopenia; N, number of study from most recent to oldest; N.R., not reported; Non-ICH, bleeding complications other than intracranial hemorrhage; Pgn, pregnancies; Ref., reference number.

a One infant presented with petechiae.

b Five infants showed minor hemorrhagic symptoms at birth.

c There were either petechiae or bruising, but severe hemorrhage was never observed.

d Symptoms were petechiae or cord bleeding.

e One of these neonates presented with cephalhematoma and petechiae.

f Three of these complications were considered serious: gastrointestinal bleeding ( *n* = 2) and bloody pericardial effusion ( *n* = 1).

### Other Neonatal Bleeds

Neonatal non-ICH bleeding was assessed in nine studies ( Table 3 ). Six of these documented bleeding events, but serious events were observed in only one of them, consisting of two gastrointestinal bleeds and a bloody pericardial effusion, in the largest cohort analyzed ( *n* = 178). 28 The meta-analysis yielded a weighted event rate for non-ICH bleeding of 0.075 (95% CI, 0.041–0.133).

### Mode of Delivery

Information regarding the mode of delivery in mothers with ITP was provided in 10 articles ( Table 4 ). Vaginal delivery was far more common (>60%) in six of them, 14 16 17 18 21 22 but two studies reported cesarean sections in more than 75% of the cases. 19 28 In one of these latter two studies, which considered 17 labors, the authors stated that, when chosen, this procedure was always performed for obstetric reasons. 19 Of the other articles that described the reasons underlying the choice of delivery mode, one reported 42 cesareans, all of which were performed in light of obstetric indications. 16 In another study, 2 of the 16 reported cesarean sections were performed because of an ITP indication, namely the concern about low neonatal platelet counts. 14 In another cohort, 18 cesareans were performed for obstetric reasons but the other seven were done because of a diagnosis of severe thrombocytopenia in the fetus. 21 In other studies, nonobstetric indications were the low platelet count (8 of 33 cesareans, platelet cutoff unspecified), 25 and prevention of vaginal delivery-caused ICH when fetal platelet counts were less than 50,000/μL in an unspecified number of cases. 23 Overall, the meta-analysis yielded a weighted event rate of 0.413 (95% CI, 0.289–0.548) for cesarean delivery from a total of 789 labors. Two articles provided information about neuroaxial anesthesia, which was noted in 25% of cases.

**Table 4:** Mode of delivery for mothers with ITP

*N*	Author	Year	Ref.	Pgn/Bb ( *n* / *n* )	Vaginal delivery, *n*	Event rate (95% CI)	Cesarean delivery, *n*	Event rate (95% CI)
1	Wegnelius et al	2018	14	75/76	59	0.787 (0.680–0.865)	16	0.213 (0.135–0.320)
2	Care et al	2018	16	107/108	65	0.607 (0.512–0.695)	42	0.393 (0.305–0.488)
3	Rezk et al	2018	17	160/155	124	0.775 (0.704–0.833)	36	0.225 (0.167–0.296)
4	Kong et al	2017	18	31/31	24	0.774 (0.596–0.888)	7	0.226 (0.112–0.404)
5	Yassaee et al	2012	19	21 a /20	3	0.143 (0.047–0.361)	17	0.810 (0.588–0.927)
6	Valat et al	1998	21	64/64	39	0.609 (0.486–0.720)	25	0.391 (0.280–0.514)
7	Christiaens et al	1997	22	68/68	46	0.676 (0.557–0.777)	22	0.324 (0.223–0.443)
8	Yamada and Fujimoto	1994	23	39/41	22	0.564 (0.407–0.709)	17	0.436 (0.291–0.593)
9	Mazzucconi et al	1993	25	63/63	30	0.476 (0.357–0.598)	33	0.524 (0.402–0.643)
10	Samuels et al	1990	28	162/162 b	38	0.216 (0.161–0.283)	124	0.705 (0.633–0.767)
Total	–	–		789/778	450	0.571 (0.417–0.712)	339	0.413 (0.289–0.548)

Abbreviations: Bb, babies; CI, confidence interval; ITP, immune thrombocytopenia; *N* , number of study, from most recent to oldest; Pgn, pregnancies; Ref., reference number.

a There were 21 pregnancies but one was aborted.

b Data correspond to index pregnancies.

### Neonatal Thrombocytopenia

Neonatal platelet counts were reported in 12 studies ( Table 5 ). Only one of them, whose cohort consisted of 31 infants, did not document cases of severe thrombocytopenia (<50,000/μL). 18 The incidence varied from 6 to 23% among the studies, and the overall estimated event rate was 0.122 (95% CI, 0.095–0.157). When considered, the lowest platelet count was always observed within 7 days of birth, often on days 2 to 4. 14 18 20 22

**Table 5:** Thrombocytopenia in neonates of mothers with ITP

*N*	Author	Year	Ref.	Pgn/Bb	Platelet count (x10 ^3^ /μL) ( *n* )			Event rate (95% CI)
				( *n* / *n* )	150–100	50–100	<50	<50 × 10 ^3^ /μL
1	Wegnelius et al	2018	14	75/69 a	N.R.	3	16	0.211 (0.133–0.316)
2	Kong et al	2017	18	31/31 b	N.R.	9	0	0.016 (0.001–0.206)
3	Yassaee et al	2012	19	21/20	N.R.	2 c	N.R.	–
4	Gandemer et al	1999	20	46/46 d	3	3	5	0.109 (0.046–0.236)
5	Valat et al	1998	21	64/64	4	4	8	0.125 (0.064–0.231)
6	Christiaens et al	1997	22	68/68 e	N.R.	12	12	0.176 (0.103–0.286)
7	Yamada and Fujimoto	1994	23	39/41	N.R.	3	5	0.128 (0.054–0.273)
8	Burrows and Kelton	1993	24	46/46	N.R.	N.R.	4	0.087 (0.033–0.210)
9	Mazzucconi et al	1993	25	63/63	7	6	4	0.063 (0.024–0.157)
10	Moutet et al	1990	26	32/32	2	1	3	0.094 (0.031–0.254)
11	Christiaens et al	1990	27	28/28	N.R.	13	4	0.143 (0.055–0.324)
12	Samuels et al	1990	28	176/178	20	20	18	0.101 (0.065–0.155)
Total	–	–		689/686	36	76	79	0.122 (0.095–0.157)

Abbreviations: Bb, babies; CI, confidence interval; ITP, immune thrombocytopenia; *N* , number of study, from most recent to oldest; N.R., not reported; Ref., reference number.

a Platelet count data were available from 69 out of 76 newborns; nadir was reached on day 2 to 4.

b Nadir was at day 3, never reaching values of <50,000/μL.

c These patients had <100,000 platelets/μL, but no additional information was given regarding whether the amounts were below or above 50,000/μL.

d Nadir was in the first week.

e Nadir was in the first week, and there were no differences in nadir counts between first and second siblings.

Predictors of neonatal thrombocytopenia were searched for in 11 of the 12 papers reporting this variable. Maternal thrombocytopenia was found to have predictive value in four cohorts. 14 21 25 26 In one of them, the neonatal platelet count correlated with the mother's nadir during pregnancy. 21 However, in a further five studies, the authors found no association between maternal and neonatal platelet counts. 18 20 22 23 24 Antiplatelet antibodies and splenectomy appeared to show more conclusive patterns. Out of five studies that analyzed the association of antiplatelet antibodies with neonatal platelets, 23 24 25 26 28 four found them to be predictive of neonatal thrombocytopenia, 23 24 25 28 especially in combination with the mother's ITP history. 28 Furthermore, one of these studies claimed that autoantibodies were the only risk factor associated with neonatal platelets. 24 Splenectomy was a risk factor for neonatal thrombocytopenia in three studies, 21 23 25 although another did not find any such association. 26 On the contrary, immunosuppressive treatment of mothers with steroids was found not to influence the neonatal platelet count in three studies, 23 26 27 even at the time of delivery. 26 Nevertheless, the study that found no association between steroid therapy in labor and neonatal platelet counts did find that the lack of maternal response to corticosteroid treatment favored the onset of thrombocytopenia in infants. 26

Other risk factors were less widely studied. Prior neonatal thrombocytopenia was found to be predictive, although this was addressed in only two articles. 14 22 In one of them, a nadir of >100,000 platelets/μL in the first sibling of a mother with ITP reduced the risk of thrombocytopenia in the second one. 22 The severity of autoimmune disorders and the mother's previous history of ITP were predictive in one 21 and two 23 28 studies, respectively. Finally, one study claimed that ITP mothers carrying the HLA DRB3* genotype were unlikely to bear a thrombocytopenic child. 20

### Maternal and Infant Mortality

The mortality of neonates and mothers was addressed by all 15 studies included in the meta-analysis ( Table 6 ). The neonatal death rate was extremely low in all studies, except for one, which reported five intrauterine fetal demises (IUFDs) and ten neonatal deaths from a total of 160 pregnancies. For this cohort, no information about the potential or indisputable association with the ITP condition of the mother was provided in addition to the main analytical results. 17 Only one IUFD 19 and one neonatal death 28 were reported in the 883 pregnancies covered by the other studies. The only neonatal death occurred after spontaneous vaginal delivery in a newborn of a mother who had circulating antiplatelet autoantibodies and who was receiving daily prednisone treatment. As mentioned previously, death resulted from an ICH, whose onset occurred when the infant had a count of 7,000 platelets/μL. This means that four newborns were recovered from an ICH episode. 22 26 28 Overall, the incidence of neonatal mortality was 1.06%. There were no maternal deaths in any of the 15 studies.

**Table 6:** Mortality in neonates and mothers with ITP

*N*	Author	Year	Ref.	Pgn/Bb ( *n* / *n* )	Neonatal death, *n* (%)	IUFD, *n* (%)	Maternal death, *n* (%)
1	Wegnelius et al	2018	14	75/76	0	0	0
2	Xu et al	2018	15	87/86	0	1	0
3	Care et al	2018	16	107/108	0	0	0
4	Rezk et al	2018	17	160/155	10 a	5	0
5	Kong et al	2017	18	31/31	0	0	0
6	Yassaee et al	2012	19	21/20	0	1	0
7	Gandemer et al	1999	20	46/46	0	0	0
8	Valat et al	1998	21	64/64	0	0	0
9	Christiaens et al	1997	22	68/68	0	0	0
10	Yamada and Fujimoto	1994	23	39/41	0	0	0
11	Burrows and Kelton	1993	24	46/46	0	0	0
12	Mazzucconi et al	1993	25	63/63	0	0	0
13	Moutet et al	1990	26	32/32	0	0	0
14	Christiaens et al	1990	27	28/28	0	0	0
15	Samuels et al	1990	28	176/178	1	0	0
Total	–	–		1,043/1,042	11 (1.06)	7 (0.67)	0

Abbreviations: Bb, babies; ITP, immune thrombocytopenia; IUFD, intrauterine fetal demise; *N* , number of study, from most recent to oldest; Pgn, pregnancies; Ref., reference number.

a Neonatal death was defined as death during the first 4 weeks after delivery.

## Discussion

Bleeding, most especially neonatal bleeding, is the main concern for physicians and mothers when the latter are diagnosed with ITP before or during pregnancy. Our findings show that primary ITP in pregnant women is rarely associated with poor outcomes.

According to our analysis, maternal bleeding is not a major concern during pregnancy. The frequency of antennal bleeding was low (18%) and most episodes were mild ( Table 1 ). For this reason, when the Gruppo Italiano Malattie EMatologiche dell'Adulto-based BAT 29 was used, more than 90% of bleeds were graded ≤2, which means that they were either petechiae, ecchymoses, or dripping with moderate blood loss. 18

The seriousness of PPH was more accurately estimated than maternal bleeding during pregnancy, as seven of the studies reported the volumes of blood loss, and the severity criterion for PPH, namely genital blood loss of ≥1,000 mL within 24 hours of delivery, is well established. 12 The rate of severe PPH varied notably between 0 and 20% in the studies that addressed this outcome 14 16 18 19 20 23 25 ( Table 2 ). But, the weighted event rate of severe postpartum hemorrhage was 0.053. The weighted event rate would cause an underestimation of bleeding risk in ITP patients. The importance of continued vigilance for bleeding complications in pregnant women with ITP should be emphasized. The authors made several arguments to explain their findings and the discrepancy with other cohorts: first, cesarean sections, which accounted for almost 40% of deliveries, are associated with greater blood loss than that occurring after a vaginal delivery; second, the amount of blood lost after labor may frequently be underestimated 30 ; and finally, physicians may subconsciously overestimate blood loss in women with thrombocytopenia.

Several studies have established that treatment must be administered to pregnant women when platelet counts fall below 30,000/μL. 6 31 32 However, what the target maternal platelet count at delivery should be remains a matter of debate.

Neonatal ICH was not a common finding in any of the 14 prospective studies that addressed the incidence of this complication 14 15 16 18 19 20 21 22 23 24 25 26 27 28 ( Table 3 ). Considering all the cohorts together, ICH occurred in only 5 of almost 900 newborns. 22 26 28 All studies were published before 1998. ICH has customarily been associated with vaginal delivery of infants with severe thrombocytopenia (<20,000 platelets/μL) and is deemed to be life-threatening. 9 10 Overall, our findings are in line with this trend, since the ratio of vaginal to cesarean deliveries was 4:1, and the platelet count was ≤7,000/μL in four of the five cases of ICH. 22 26 28 Given these data, tools capable of accurately estimating the platelet count of infants at birth would be extremely helpful. Unfortunately, methods such as scalp sampling or percutaneous umbilical blood sampling are no longer used for this purpose. Platelet numbers in collected samples are not necessarily correlated with those subsequently determined in the newborns, and these procedures are associated with an unacceptable risk of hemorrhage and/or prematurity. 32

Platelet counts >50,000/μL are deemed safe for a normal vaginal delivery as well as for a cesarean section, provided that the platelet threshold is suitable for neuraxial anesthesia. 33 Overall, the proportion of cesarean deliveries among more than 1,000 ITP mothers exceeded 40% 14 16 17 18 21 22 ( Table 4 ). Obstetric issues were by far the most frequent reason why this option was chosen. 16 19 Low maternal platelet counts 25 or the concern about a low neonatal platelet count 14 were among the ITP-related causes.

Ten studies reported neonatal platelet counts <50,000/μL in fewer than 20% of infants, 18 20 21 22 23 24 25 26 27 28 while another study documented an incidence of 21% 14 ( Table 5 ). Predictors of severe thrombocytopenia were not readily identified in primary studies, but maternal platelet autoantibodies and splenectomy were frequent associations.

Our study has limitations. First, very few prospective studies specifically focused on ITP management of pregnant women. Second, there is a substantial temporal gap between 1999 and 2012, a period during which we did not find any studies that fulfilled all the inclusion criteria. Furthermore, nine of the 15 selected studies were published before 2009 (20–28). This means that they did not follow the recommendations regarding the standardization of terminology, definitions, and outcome criteria that were proposed by an international working group in the same year. 1 Discrepant criteria regarding those topics further challenged the analyses. Only 3 of the 15 selected studies covered the main objectives addressed by this review. 14 18 25 Moreover, the size of the cohorts was often rather small. Therefore, the results of the meta-analyses, though informative, are often of less quality than desirable. Furthermore, the quality of reporting of bleeding was low in most of the selected studies. Finally, the influence of the treatment of ITP mothers on outcomes, with respect to the conditions that should prompt initiation and the drugs that should be used as first-line, could not be included in the meta-analysis, since this topic has not been adequately addressed in the majority of studies.

## Conclusion

Our study is the most comprehensive summary of bleeding frequency and severity in the ITP pregnancy literature. Primary ITP in pregnant women is rarely associated with poor outcomes. It seems reasonable to inform ITP women that their condition is not a contraindication to pregnancy.
