# Supplementary material for: Bleeding Complications Associated with Pregnancy with Primary Immune Thrombocytopenia: A Meta-Analysis
Source: TH Open. 2022 Aug 29;6(3):e230–7. doi: 10.1055/a-1837-7581 (PMC9423940; doi:10.1055/a-1837-7581)
Supplement: Supplementary file 1 — Supplementary Material [file 10-1055-a-1837-7581-s210063.pdf]

Table S1 Aims, BATs and/or bleeding monitoring procedures, and objectives of this review addressed by the selected studies

Author [ref]	Year	Aims	BAT / Bleeding assessment	Review objectives covered					
				Pregnancy bleeding	Delivery mode	Postpartum hemorrhage	Neonatal ICH	Neonatal TPN	Mortality
Wegnelius et al[14]	2018	To assess a routine for treatment of ITP in pregnancy	No BAT<br>At pregnancy: anatomical sites<br>At delivery: blood loss >1,000 mL	X	X	X	X	X	X
Xu et al[15]	2018	To evaluate the response rate of glucocorticoid and/or immunoglobulin therapy in severe thrombocytopenia and the incidence of maternal complications	No BAT<br>ICH assessment	–	–	–	X	–	X
Care et al[16]	2018	To quantify the incidence of severe ITP in pregnancy in the UK, determine current treatment strategies, and establish maternal and neonatal morbidity and mortality associated with severe ITP in pregnancy	No BAT<br>Bleeding episodes in antenatal period, PPH	X	X	X	X	–	X
Rezk et al[17]	2018	To assess prospectively maternal and fetal outcome in women with ITP who undergone earlier splenectomy, compared with women on medical therapy.	No BAT<br>Bleeding episodes in antenatal period, PPH	X	X	–	–	–	X
Kong et al[18]	2017	To determine the safety and efficacy of rhTPO for ITP management during pregnancy	BAT: severity scoring system reported by the GIMEMA ITP Working Party [28]<br>Bleeding was assessed on days 1 and 14 of rhTPO therapy	X	X	X	X	X	X
Yassaee et al[19]	2012	To document the course of disease and the outcome of pregnancy in pregnant women with ITP	No BAT<br>Bleeding episodes in antenatal period, PPH	–	X	X	X	X	X
Gandemer et al[20]	1999	To determine if identification of platelet-specific autoantibodies in association with genetic markers such as HLA typing in pregnant women with isolated thrombocytopenia, ITP or ITP history could predict neonatal thrombocytopenia	No BAT<br>Bleeding episodes in antenatal period, PPH	X	–	X	X	X	X

(*Continued*)

Table S1 (*Continued*)

Author [ref]	Year	Aims	BAT / Bleeding assessment	Review objectives covered					
				Pregnancy bleeding	Delivery mode	Postpartum hemorrhage	Neonatal ICH	Neonatal TPN	Mortality
Valat et al[21]	1998	To evaluate the relationships between the course of the maternal disease before and during pregnancy and the risk of severe fetal thrombocytopenia	No BAT<br>ICH assessment	–	X	–	X	X	X
Christiaens et al[22]	1997	To estimate the predictive value of the first neonatal platelet count to the outcome of the second neonate in women with ITP	No BAT<br>ICH assessment	–	X	–	X	X	X
Yamada and Fujimoto[23]	1994	To evaluate the influence of therapies for maternal ITP on fetal passive ITP	No BAT<br>Bleeding episodes in antenatal period, PPH	–	X	X	X	X	X
Burrows and Kelton [24]	1993	To study the risks associated with fetal thrombocytopenia among the infants born to a cohort of women at a tertiary hospital, a proportion of whom had ITP	No BAT<br>ICH assessment	–	–	–	X	X	X
Mazzucconi et al[25]	1993	To evaluate whether maternal risk factors, when present in the third trimester, can shed light on predicting neonatal thrombocytopenia	No BAT<br>Bleeding episodes in antenatal period, PPH	X	X	X	X	X	X
Moutet et al[26]	1990	To determine maternal characteristics with predictive value for the fetal platelet count	No BAT<br>ICH assessment	–	–	–	X	X	X
Christiaens et al[27]	1990	To determine the efficacy of antenatal low-dose oral betamethasone in preventing neonatal thrombocytopenia and/or bleeding in infants of mothers with ITP	No BAT<br>ICH assessment	–	–	–	X	X	X
Samuels et al[28]	1990	To estimate the risk of neonatal thrombocytopenia and hemorrhage in infants born to mothers with ITP	No BAT<br>ICH assessment	–	X	–	X	X	X

Abbreviations: BAT, bleeding assessment tool; GIMEMA, Italian Adult Haematological Diseases Group; HLA, human leucocyte antigen; ITP, immune thrombocytopenia; NR, not reported; PPH, postpartum hemorrhage; Ref., reference number; rhTPO, recombinant human thrombopoietin; TPN, thrombocytopenia.

 © 2022. The Author(s).
